# Dynamic Deconstructive Psychotherapy for Suicidal Adolescents: Effectiveness of Routine Care in an Outpatient Clinic

**DOI:** 10.3390/ijerph21070929

**Published:** 2024-07-16

**Authors:** Rebecca J. Shields, Jessica P. Helfrich, Robert J. Gregory

**Affiliations:** Department of Psychiatry and Behavioral Sciences, SUNY Upstate Medical University, Syracuse, NY 13210, USA; shieldsr@upstate.edu (R.J.S.); helfricj@upstate.edu (J.P.H.)

**Keywords:** adolescent, suicide, depression, psychotherapy, public health

## Abstract

Suicidal behavior and demand for services have been increasing in adolescents. Many of the current treatments are focused on symptom mitigation, crisis management, and safety planning; however, few are aimed at remediating underlying vulnerabilities that may be contributing to suicide risk. Dynamic Deconstructive Psychotherapy (DDP) has been found to be effective for suicidal adults but has never been studied for adolescents. The present study examined real-world outcomes of 65 suicidal adolescents, aged 13–17 years, receiving weekly DDP in an outpatient clinic. The primary outcome was change in suicide ideation from baseline to 6 months of treatment as assessed by the Suicide Ideation Subscale of the Columbia Suicide Severity Rating Scale. In intent-to-treat analyses, suicide ideation significantly decreased over the 6 months with a large treatment effect (*d* = 1.19). Secondary measures, such as suicide attempts, self-harm, depression, anxiety, disability, self-compassion, and inpatient utilization, also improved significantly. Among the 42 adolescents (65%) who completed at least 6 months of treatment, suicide attempts decreased by 84%. DDP may be effective in reducing suicide ideation and other risk factors in suicidal adolescents and may be cost-effective given reduced inpatient utilization. These initial promising findings warrant further research and development.

## 1. Introduction

Rates of suicide and suicidal behavior among adolescents are on the rise. From 2009–2018, suicide rates among youths aged 14–18 increased by 62% [1]. According to 2020 data from the U.S. Centers for Disease Control and Prevention (CDC), suicide is the second leading cause of death in the U.S. in the 10–14 and 25–34 age ranges, and the third leading cause of death in the 15–24 age range [2]. Worldwide, suicide was the third leading cause of death for females aged 15–19 and the fourth leading cause of death for males in 2019 [3].

Although many adolescents die by suicide each year, a great many more are at risk. According to the CDC’s 2021 Youth Risk Behavior Survey in the U.S., 42% of students (14–18 years old) felt persistently sad or hopeless, 22% seriously considered attempting suicide, and 10% have attempted suicide [4]. In the decade between 2009 and 2019, the proportion of pediatric hospitalizations in the U.S. for reasons of mental illness rose by 72% [5] and suicide-related visits to emergency departments rose five-fold [6]. In light of these trends, the U.S. Surgeon General declared there to be a youth mental health crisis in December 2021 and called on the nation to respond [7].

Given this crisis of adolescent suicide in the U.S. and in much of the world, it is surprising how few evidence-based psychotherapies have been developed thus far for suicidal adolescents. The best evidence is for a modified version of dialectical behavior therapy (DBT-A). A meta-analysis of controlled trials indicated small to moderate effects for reducing self-harm and suicide ideation compared to control groups, and large pre-post effects for all outcomes [8]. A more recent randomized controlled trial (RCT) of DBT-A for adolescents with bipolar spectrum disorders indicated reduced suicide attempts compared to the control psychotherapies [9]. Some other individual psychotherapies showing promising results for suicidal adolescents in RCTs include mentalization-based treatment [10] and cognitive behavior therapy [11]. However, subsequent studies of these two treatments have shown mixed results [12,13].

With so few available evidence-based psychotherapies for suicidal adolescents, there is a need for continued development of alternative psychotherapy models, especially for those adolescents who do not respond to the gold standard DBT-A, or who do not have that treatment available in their community. With this in mind, we attempted to apply a different psychotherapy model for this population called Dynamic Deconstructive Psychotherapy (DDP). We present the empirical results of our clinical experience with DDP in this paper.

DDP is a manual-based psychodynamic therapy involving individual weekly sessions for up to 12 months [14]. It was initially developed for severe and treatment-resistant cases of borderline personality disorder (BPD) in adults and was shown to be effective for BPD symptoms in two randomized controlled trials [15,16,17] and a naturalistic cohort comparison study [18]. An analysis of secondary outcomes of these trials indicated efficacy for depression and suicidal behaviors. A more recent cohort comparison study evaluated the efficacy of DDP when applied to suicidal adults attending a specialized high-risk treatment program [19]. It reported a large reduction in rehospitalizations over the first six months of treatment compared to the control group, and large reductions in depression and suicide ideation.

The present study adds to the research literature on DDP as the first empirical evaluation of DDP for suicidal adolescents. The study takes place in a real-world outpatient clinic and retrospectively evaluates whether high-risk adolescents who received treatment with DDP in the clinic were able to achieve suicide risk reduction on standardized measures. Our a priori hypothesis was that suicide ideation in high-risk adolescents receiving DDP would significantly decrease over the first 6 months of treatment.

## 2. Materials and Methods

### 2.1. Setting

The Psychiatry High-Risk Program (PHRP) was established in 2017 in response to a community-led taskforce recommendation for a specialized outpatient treatment program for high-risk adolescents and young adults. The PHRP provides up to 12 months of comprehensive treatment for youth and young adults ages 14 through 40 years of age who are struggling with thoughts of suicide. Patients are referred to the PHRP via multiple referral sources, including but not limited to emergency departments, psychiatric hospitals, outpatient psychiatric clinics, and self-referrals in the general community. The PHRP does not admit patients who have a primary psychotic disorder, major developmental disability, or other circumstances that limit the individual’s ability to engage in weekly psychotherapy. Each patient undergoes a comprehensive psychiatric evaluation and may be provided with medication management, family therapy, and group therapy according to clinical indications and the patient’s willingness to engage in them.

### 2.2. Participants

This was a retrospective naturalistic study examining the outcomes of consecutive suicidal adolescents admitted for treatment in the PHRP. For the purposes of this study, we included all adolescents who had been admitted to the PHRP between June 2020 and July 2023 who had evidence of active suicide ideation as indicated by a Columbia Suicide Severity Scale (C-SSRS) [20] score on the Suicide Ideation Subscale of ≥2. A total of 65 adolescents met these study criteria.

The demographic characteristics of the sample are outlined in Table 1. The mean age of study participants was 15.71 ± 1.10 years (range: 13–17 years). Participants were predominantly female sex (*n* = 55; 85%), cisgender (*n* = 44; 68%), White non-Hispanic race/ethnicity (*n* = 58; 89%), and had private insurance (*n* = 39; 60%).

The clinical diagnoses of participants assigned by PHRP psychiatric practitioners are outlined in Table 2. Borderline personality disorder, major depressive disorder, generalized anxiety disorder, and post-traumatic stress disorder were the most common diagnoses. The mean Adverse Childhood Experiences score was 3.88 ± 2.69, suggesting substantial childhood adversity in this population.

All but one of the adolescents in our sample (98%) described chronic psychiatric symptoms or behaviors of at least 2 years duration. Thirty of the adolescents (46%) had been tried on 3 or more antidepressants before entering the program, suggesting a high level of treatment resistance in this population. A total of 63 adolescents (97%) endorsed a history of self-harming behavior within the past 2 years, and 51 (78%) endorsed self-harming within the past 30 days. Sixty-two adolescents (95%) in our sample endorsed at least one lifetime suicide attempt, with a median of 7 attempts (Interquartile range = 3–17). Thirty-six adolescents (55%) endorsed at least one suicide attempt within the 90 days prior to entering the program.

### 2.3. Treatments

Weekly individual psychotherapy with DDP was provided by 10 licensed providers, including 5 clinical social workers, 3 attending-level child and adolescent psychiatrists, an attending-level general psychiatrist, and a child psychologist. All but one of the providers had at least two years of training and experience in DDP and advanced certification in this modality, and that provider received weekly individual case consultations. All providers were also required to participate in weekly small peer consultation groups of no more than six providers to discuss challenging cases and maintain fidelity to the DDP treatment model. Importantly, all team members, even prescribers, are trained in DDP theory and methods. The training provides a common language and understanding of cases, minimizes splitting, and facilitates case discussion and feedback.

Treatment with DDP begins with a prolonged intake process to: (1) gain a deeper understanding of the strengths, vulnerabilities, and relational patterns of the adolescents and their families; (2) establish a safety plan; (3) develop a formulation that can be presented to the parents and adolescents that they can endorse; (4) lay out a pathway to recovery that injects hope and clarifies the goals and tasks of the treatment; and (5) review the minimum commitments necessary for recovery to instill a sense of ownership in their own health and recovery.

The full treatment model of DDP is in the training manual, available on the DDP website (www.upstate.edu/ddp; accessed on 20 March 2023) and summarized in an earlier paper [14]. In brief, the overarching goal is to facilitate the development of a more coherent sense of self through remediation of three sets of deficits in the brain’s emotion processing system. There are three sets of techniques for each of these deficits called *Association*, *Attribution*, and *Alterity* techniques. Association techniques facilitate the client’s ability to verbalize and sequence recent interpersonal experiences or maladaptive behaviors, thereby activating and remediating autobiographical memory and affect labeling [21]. Attribution techniques involve questioning and integrating alternative or opposing meanings to those experiences. Alterity techniques are the most unique aspects of DDP in that they rely on intersubjective experiences between the client and the therapist to foster a capacity for authentic relatedness, self-soothing, and self-compassion.

Borrowing from Emile Durkheim’s theory of *Egocide* and the Interpersonal Theory of Suicide [22,23], DDP views vulnerability to suicide in adolescents as secondary to the sense of being *stuck alone with overwhelming pain*. This simple phrase brings together elements of hopelessness, alienation, disconnection, and a limited ability to process emotionally painful experiences. DDP targets each of these elements by injecting hope of recovery, fostering authentic relatedness, improving self-compassion, and remediating the emotion processing system through activating autobiographical memory of recent emotion-laden interactions and labeling specific emotions.

Other treatment components at the PHRP are used to supplement and enhance the work of individual psychotherapy. Medications can relieve symptoms and may also sometimes help patients make better use of psychotherapy. Fifty-nine participants (91%) were taking prescribed psychotropic medications at the time of entry into the PHRP, with an average of 2.40 ± 1.41 medications per participant.

Family therapy and group therapy are offered in the PHRP, usually by a clinician other than the individual therapist. These treatments are utilized to help increase the capacity for authentic relationships within and outside the family, thereby decreasing the adolescent’s sense of isolation and alienation. Family therapy is strongly encouraged for adolescents, though many decline. Monthly psychoeducation seminars for the adolescents and family members provide better knowledge of risk factors, suicide prevention strategies, and ways to enhance health and wellness. In-house case management services alleviate some of the social stresses on families and help to coordinate care. In addition, the individual therapist reviews the results of quarterly outcome measures with both the adolescent and the parents, which is helpful for remotivating the patient and family engagement in treatment.

### 2.4. Measures

An important component of DDP is tight quality assurance, which includes the administration of self-rated outcome measures at the time of intake as well as every 3 months that the adolescent is in the PHRP. In addition, at the time of admission, a trained Bachelor’s-level testing coordinator administers the C-SSRS. These measures are entered into an Excel spreadsheet by the intake coordinator and provide a way to monitor program participants’ outcomes.

A priori to analyses, for our primary outcome, we used the change in the C-SSRS Suicide Ideation Subscale from baseline to 6 months. This measure involves a series of five Yes/No questions progressing in severity: (1) passive wishes to be dead, (2) thoughts of killing oneself, (3) thinking about a method to kill oneself, (4) having some intent to carry out suicide, and (5) having a specific plan with intent. Depending on the number of “Yes” responses to these five items, the suicide ideation score ranges from 0 to 5. Responses to this measure have been shown to predict future suicide attempts [24].

Secondary measures included suicide attempts, as assessed with the Suicidal Behavior Subscale of the C-SSRS. This measure encompasses actual suicide attempts, interrupted attempts, and aborted attempts. Secondary symptom measures included the Patient Health Questionnaire-9 (PHQ-9) for depression severity, and General Anxiety Disorder-7 (GAD-7) for anxiety severity [25]. We separately examined item 9 of the PHQ-9 as a secondary measure of suicide ideation frequency since the endorsement of this item is predictive of suicide attempts and completions [26]. Functional impairment was self-assessed with the 3-item Sheehan Disability Scale [27] and self-compassion with the 12-item Self-Compassion Scale [28]. Self-harm was tracked with a single question on a questionnaire: “How many days in the past month did you try to harm yourself by cutting, overdose, puncturing, burning, or smothering?”.

In addition, for the purposes of this study, a review of the electronic medical records was performed by two of the investigators (R.J.S. and J.P.H.) to record the number of outpatient visits to the PHRP, visits to the emergency department (ED), psychiatric hospitalizations, and hospital days. The timeline of the review was for the period from 3 months before admission to 6 months after admission to the PHRP. From this data, we were able to compare inpatient utilization during the 90-day interval before admission to the PHRP to inpatient utilization during the 90-day interval before the 6-month mark of treatment.

### 2.5. Statistical Analysis

We imported the data into the Statistical Package for Social Sciences, version 28.0.1.0 (SPSS Inc., Chicago, IL, USA) for statistical analysis of the intent-to-treat population (*n* = 65). As most outcome measures at baseline and 6 months violated the assumption of normality using the Shapiro–Wilk test, we analyzed our data with the Wilcoxon Signed-Ranks test, a nonparametric alternative to the paired-samples *t*-test. We used the Wilcoxon Signed-Ranks test to examine the statistical significance of pre-post differences in the primary outcome measure (suicide ideation) and all secondary measures between baseline and 6 months of DDP treatment. All tests were conducted as two-tailed with a significance level of *p* < 0.05.

Missing data analysis revealed that primary and secondary variables were missing responses for 13 participants (20%) at the 3-month measurement and 23 participants (35%) at the 6-month measurement (18% missing data points in total). We assessed the missing data pattern with Little’s Missing Completely at Random test, which indicated that the data were completely missing at random (χ^2^ = 24.127, *df* = 24, *p* = 0.454). Missing data points of the primary and secondary outcome measures at 3 months and 6 months were imputed with multiple imputations. We created 50 imputed datasets and used the pooled estimates for the Wilcoxon Signed-Ranks test. All means reported in this paper were unadjusted by multiple imputations. Furthermore, since there were no missing data for emergency department and inpatient utilization chart reviews, no imputation was necessary for these measures.

We utilized two measures of treatment effects for the outcome measures in the intent-to-treat sample. We used *r_c_* to estimate the size of the pre–post treatment effects. We calculated *r_c_* by dividing the *z* value obtained from the Wilcoxon Signed-Ranks test by the square root of *N* [29]. We also added Cohen’s *d* as a measure of treatment effects since it is more widely used and can facilitate direct comparisons between the treatment effects of DDP and those of other treatments in this population. It was calculated as the difference in mean scores at pretreatment and at 6 months divided by the pooled standard deviation [30]. We interpreted *r_c_* values of 0.1 as a small effect size, 0.3 as a medium effect size, and 0.5 as a large effect size. We interpreted Cohen’s *d* values of 0.2, 0.5, and 0.8 as a small, medium, and large effect size, respectively.

All data used in post-hoc analyses were assessed using the Shapiro–Wilk test for normality. For distributions that significantly deviated from normality, non-parametric tests were chosen when conducting the analyses.

## 3. Results

### 3.1. Primary Outcome

Forty-two (65%) of the adolescents who had been admitted to the PHRP completed at least 6 months of treatment. The average number of individual therapy sessions in the intent-to-treat sample was 18.17 ± 6.70. Twenty-six adolescents (40%) had at least one family therapy session and 15 adolescents (23%) participated in group therapy. For adolescents who stayed in treatment for at least 6 months, the average number of individual therapy sessions was 21.62 ± 2.88, and *n* = 22 (52%) participated in family therapy and *n* = 13 (31%) in group therapy.

As indicated in the first row of Table 3, suicide ideation decreased by 44% over 6 months of treatment, displaying a large effect. The Cohen’s *d* value was also large (*d* = 1.19 [0.82, 1.56]). In intent-to-treat analysis, this change was statistically significant. Of those who completed at least 6 months of treatment, *n* = 23 (55%) achieved a clinical response, defined arbitrarily by at least a 50% decrease in the C-SSRS suicide ideation score.

Post-hoc exploratory analyses were performed to assess the effect of four demographic variables on change in suicidal ideation after six months of treatment, i.e., age, gender identity, race/ethnicity, and insurance. The results of Pearson’s product–moment correlation indicated that there was no statistically significant association between age and change in suicide ideation, *r*(63) = 0.152, *p* = 0.227. Independent sample *t*-tests revealed no statistically significant difference in the change in suicide ideation between cisgender participants (*M* = 1.37, *SD* = 1.61) and transgender/non-binary participants (*M* = 1.54, *SD* = 1.39); *t*(63) = 0.418, *p* = 0.678. Likewise, there was no statistically significant difference between White/non-Hispanic participants (*M* = 1.54, *SD* = 1.53) and those with other racial/ethnic identities (*M* = 0.47, *SD* = 1.26); *t*(63) = −1.763, *p* = 0.083, though a trend was evident. We also found no statistically significant difference between participants with government-sponsored health insurance (*M* = 1.51, *SD* = 1.38) and those with private insurance (*M* = 1.36, *SD* = 1.64); *t*(63) = -.383, *p* = 0.703.

Twelve adolescents were discharged in the first 3 months (18%), and a further 11 adolescents in the second quarter. Patients are discharged from the program before 6 months if they repeatedly violate their initial commitments, most commonly by not attending weekly sessions, or if they decide to end treatment. A post-hoc analysis indicated that change in suicide ideation in the first 3 months of treatment was not significantly related to ending treatment in the following 3 months (Mann-Whitney *U* = 195.50, *p* = 0.495). This indicates that patients did not drop out due to a lack of treatment response.

### 3.2. Secondary Outcomes

All secondary outcomes showed statistically significant improvement between baseline and 6 months, including measures of depression, anxiety, self-harm, suicide attempts, as well as disability and self-compassion. Treatment effects were medium to large. Cohen’s *d* effect sizes were consistent in magnitude with *r_c_* effect sizes, ranging from 0.53 to 1.18. Notably, suicide attempts as measured by the C-SSRS decreased by 67% in the intent-to-treat sample and by 84% among those who completed at least 6 months of treatment.

### 3.3. Utilization

Forty of the adolescents (62%) had visited the ED for psychiatric issues in the 3 months preceding admission to the PHRP, but only 15 adolescents (23%) in the first 3 months (90 days) following admission to the PHRP. Twenty-five adolescents (38%) were psychiatrically hospitalized in the 3 months before admission to the PHRP, but only 5 (8%) were re-hospitalized in the first 3 months following admission.

**Figure 1:**
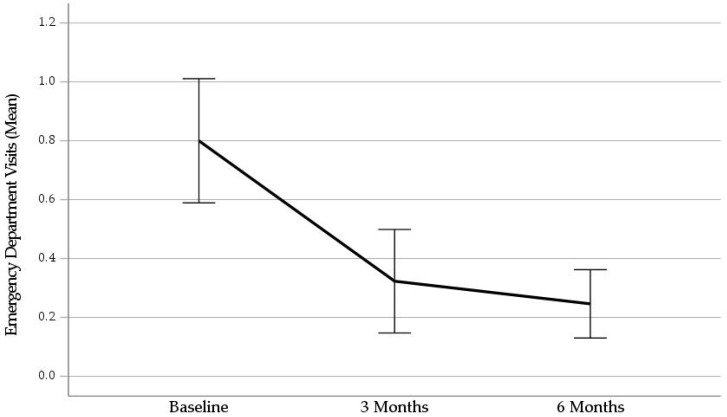
Mean Number of Emergency Department Visits Over the Prior 90 Days. Note. Error bars represent 95% confidence intervals.

**Figure 2:**
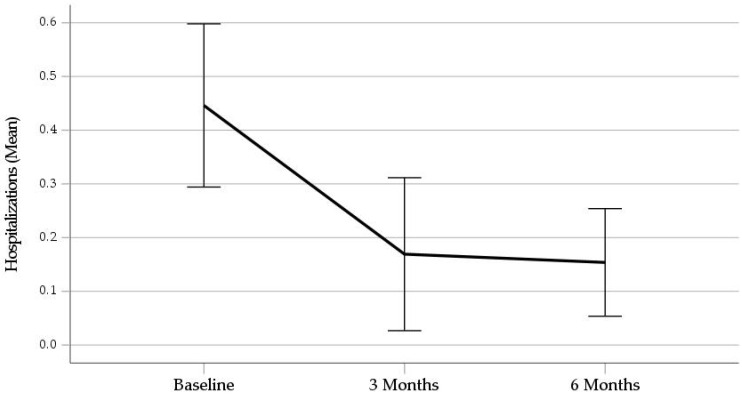
Mean Number of Hospitalizations Over the Prior 90 Days. Note. Error bars represent 95% confidence intervals.

The second 3 months of the study period were characterized by significant reductions in both the mean number of visits to the ED (*Z* = −4.71, *p* < 0.001) and the mean number of psychiatric hospitalizations (*Z* = −3.40, *p* < 0.001), as compared to the 90-day interval preceding admission to the PHRP in intent-to-treat analyses. The pre and post means with 95% confidence intervals are displayed graphically in Figure 1 and Figure 2. The mean number of visits to the ED dropped by 69% and the mean number of hospitalizations dropped by 67%. The mean number of hospital days decreased from 6.23 ± 12.56 at pretreatment to 1.65 ± 5.50 at 6 months, with an average net savings of about 4.5 hospital days per adolescent.

## 4. Discussion

This is the first published paper on the efficacy of DDP as a treatment for suicidal adolescents. DDP has already been studied in adults and has shown to be helpful for various psychiatric symptoms, including but not limited to BPD, depression, and suicide ideation [15,16,17,18,19]. However, it has never been formally studied in adolescents. The primary finding of our study is that suicide ideation in high-risk adolescents decreased with the use of DDP applied weekly over 6 months. The treatment effect of DDP in the intent-to-treat sample was large and statistically significant. This finding is consistent with our hypothesis and with adult studies of DDP. The large treatment effect is especially notable given the severity of our study sample, characterized by chronic illness, numerous lifetime suicide attempts, and a high rate of non-response to multiple antidepressant medication trials.

A second major finding from our study is that adolescents receiving treatment with DDP not only experienced reductions in suicide ideation but also demonstrated broad-based and statistically significant improvements across other outcome variables that could contribute to suicide risk, with medium to large effects. Depression, anxiety, self-harm, suicide attempts, self-compassion, and functioning all significantly improved. Improvements in self-compassion and functional impairment are especially important targets for a recovery-based treatment since they suggest more fundamental changes to underlying vulnerabilities to suicide, rather than merely symptom mitigation and crisis management. Further research is needed to determine whether these changes are long-lasting and to determine the mediators and moderators of treatment response.

Of note, about a third of participants dropped out during treatment. Difficulty engaging suicidal adolescents in treatment has been noted in the literature, with attrition rates as high as 77% [31]. The reasons for this are unclear. In the present study, lack of treatment response was not a significant predictor of dropout. Treatment completion rates vary widely in research studies of suicidal adolescents but are generally comparable to the present study [32]. These findings suggest a need to find more effective ways to keep suicidal adolescents engaged in treatment.

The third important finding from our study is that adolescents treated with DDP demonstrated significant reductions in inpatient utilization. Visits to the ED and psychiatric hospitalizations decreased by almost 70%. The findings in the present study are consistent with a cohort comparison study of suicidal young adults being treated with DDP, which indicated marked reductions in inpatient utilization as compared to usual care, with savings of over 6 hospital days per patient over the first 6 months of treatment [19].

Improvement in inpatient utilization is especially important given the current crisis of suicidal youth flooding the ED for mental health conditions and suicide attempts. Between 2012 and 2016, hospitals in the U.S. experienced a 51% increase in child and adolescent visits to the ED for mental health conditions, compared to only 13% for non-mental health conditions [33]. ED visits accelerated in the U.S. during the pandemic, especially among adolescent females, with visits for suspected suicide attempts in that cohort increasing by 51% between 2019 and 2021 [34]. Rehospitalization is not only costly but may contribute to an increased risk of future suicide attempts [35]. DDP shows promise as one possible treatment for breaking the cycle of illness and suicide among adolescents and helping to keep them out of the ED and hospitals. Further research is needed to determine the cost-effectiveness of DDP as compared to usual care and to examine long-term outcomes.

A limitation of this study is the lack of a control group. It is unclear whether DDP is more effective than usual care for suicidal adolescents, and whether the findings in the present study can be generalized to adolescents in other regions. None of the demographic variables we measured were significant predictors of improvement in suicide ideation, thereby supporting the potential generalizability of our findings to diverse populations. However, the relative homogeneity of our patient population, especially regarding race/ethnicity, suggests the need for further study. Network analysis may offer potential as a method to better understand interactions between demographic variables and other possible moderators or mediators of treatment response, such as motivation, to better guide treatment decisions [36].

Another limitation is that there is a potential self-selection bias of adolescents willing to participate in the treatment. Nevertheless, the large and significant treatment effects in this challenging adolescent population support the feasibility of a recovery-based approach to suicide prevention. Moreover, the decreased ED visits and hospitalizations suggest that treatment with DDP may be cost-effective, and this warrants further investigation. Overall, the findings from this study are a promising step towards the larger goal of helping suicidal adolescents to have greater access to care, particularly for those who have already tried other more fully investigated treatments such as DBT-A.

## 5. Conclusions

Adolescent suicide has become a growing and concerning issue in many areas of the world, and yet there are few effective treatments and access is limited. DDP has demonstrated efficacy in decreasing suicide ideation in adults but has never been formally studied in the adolescent population; the present paper is the first published study of DDP for this population. The real-world findings in this study suggest that DDP might be effective in decreasing suicide risk and addressing underlying vulnerabilities to suicide in high-risk adolescents. These initial promising findings warrant further research and development.

## Figures and Tables

**Table 1:** Sociodemographic characteristics of participants.

	Participants	
Sociodemographic Characteristics	*n*	%
Age		
13–15	26	40
16	20	31
17	19	29
Sex Assigned at Birth		
Male	10	15
Female	55	85
Gender Identity		
Cisgender	44	68
Transgender	6	9
Non-Binary	15	23
Race		
American Indian or Alaska Native	1	2
Asian	2	3
Black or African American	1	2
Native Hawaiian or Other Pacific Islander	0	0
White	61	94
Ethnicity		
Hispanic or Latino	3	5
Not Hispanic or Latino	62	95
Insurance		
Government Insurance	26	40
Private Insurance	39	60

Note. *N* = 65.

**Table 2:** Clinical diagnoses of participants.

	Participants	
Clinical Diagnoses	*n*	%
Attention-deficit/hyperactivity disorder	7	11
Autism spectrum disorder	2	3
Other neurodevelopmental disorders	1	2
Major depressive disorder	61	94
Persistent depressive disorder	4	6
Other mood disorders	3	5
Panic disorder	4	6
Generalized anxiety disorder	31	48
Other anxiety disorders	4	6
Obsessive-compulsive disorder	8	12
Post-traumatic stress disorder	35	54
Dissociative identity disorder	1	2
Anorexia nervosa	10	15
Bulimia nervosa	7	11
Binge-eating disorder	3	5
Other feeding or eating disorders	3	5
Gender dysphoria	7	11
Alcohol use disorder	5	8
Cannabis use disorder	17	26
Nicotine use disorder	4	6
Borderline personality disorder	50	77

Note. *N* = 65.

**Table 3:** Primary and secondary outcomes over 6 months of treatment.

Measure	Baseline *M* (*SD*)	3 Months *M* (*SD*)	6 Months *M* (*SD*)	*Z*	*p*	* r * *_c_*
C-SSRS Suicide Ideation	3.26 (1.06)	2.25 (1.41)	1.74 (1.62)	−5.52	<0.001	0.68
C-SSRS Suicide Attempts	1.38 (2.34)	0.46 (1.04)	0.26 (0.91)	−3.64	<0.001	0.45
PHQ-9 Depression	19.86 (4.84)	16.98 (5.17)	14.12 (6.39)	−5.69	<0.001	0.71
PHQ-9 Suicide Ideation	2.11 (1.00)	1.48 (1.04)	1.00 (1.06)	−5.67	<0.001	0.70
Self-Harm Behaviors	5.51 (7.40)	1.52 (2.12)	0.86 (1.89)	−5.23	<0.001	0.65
GAD-7 Anxiety	15.71 (4.61)	13.98 (4.56)	11.79 (5.24)	−4.92	<0.001	0.61
Sheehan Disability Scale	6.97 (1.77)	6.63 (1.84)	5.37 (2.72)	−3.93	<0.001	0.49
Self-Compassion Scale	2.07 (0.54)	2.40 (0.65)	2.68 (0.65)	−5.96	<0.001	0.74

Note. *M* = mean. *SD* = standard deviation. *Z* = Wilcoxon Signed Ranks Test *Z*-score. *p* = probability value. *r_c_ =* Z/√N.

## Data Availability

The dataset from this study is not publicly available since U.S. federal laws prohibit the sharing of private health information by healthcare providers. However, the corresponding author will make available a de-identified dataset upon request to researchers who wish to conduct further analyses on that dataset as part of a study approved by the Institutional Review Boards of the respective sending and receiving institutions.
